# Operando Electrochemical Liquid Cell Scanning Transmission Electron Microscopy Investigation of the Growth and Evolution of the Mosaic Solid Electrolyte Interphase for Lithium-Ion Batteries

**DOI:** 10.1021/acsnano.3c06879

**Published:** 2023-10-13

**Authors:** Walid Dachraoui, Robin Pauer, Corsin Battaglia, Rolf Erni

**Affiliations:** † Electron Microscopy Center, Empa—Swiss Federal Laboratories for Materials Science and Technology, Überlandstrasse 129, 8600 Dübendorf, Switzerland; ‡ Materials for Energy Conversion, Empa—Swiss Federal Laboratories for Materials Science and Technology, Überlandstrasse 129, 8600 Dübendorf, Switzerland; § Departement of Information Technology and Electrical Engineering—ETH Zürich, Gloriastrasse 35, 8092 Zürich, Switzerland; ∥ Institute of Materials−EPFL, Station 12, 1015 Lausanne, Switzerland; ⊥ Departement of Materials—ETH Zürich, Wolfgang-Pauli-Strasse 10, 8049 Zürich, Switzerland

**Keywords:** in situ S/TEM, electrochemical liquid cell TEM, Li-ion batteries, solid electrolyte interphase, structural growth, interphase formation

## Abstract

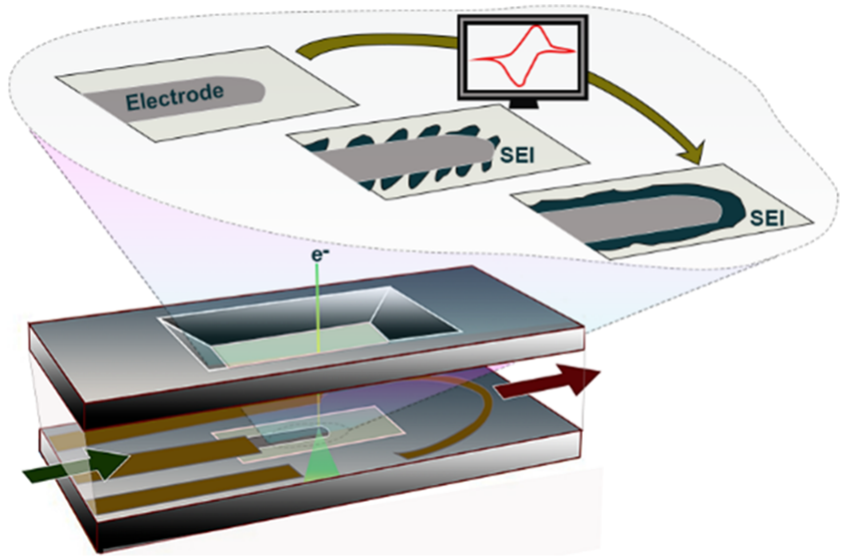

The solid electrolyte interphase (SEI) is a key component of a lithium-ion battery forming during the first few dischage/charge cycles at the interface between the anode and the electrolyte. The SEI passivates the anode–electrolyte interface by inhibiting further electrolyte decomposition, extending the battery’s cycle life. Insights into SEI growth and evolution in terms of structure and composition remain difficult to access. To unravel the formation of the SEI layer during the first cycles, operando electrochemical liquid cell scanning transmission electron microscopy (ec-LC-STEM) is employed to monitor in real time the nanoscale processes that occur at the anode–electrolyte interface in their native electrolyte environment. The results show that the formation of the SEI layer is not a one-step process but comprises multiple steps. The growth of the SEI is initiated at low potential during the first charge by decomposition of the electrolyte leading to the nucleation of inorganic nanoparticles. Thereafter, the growth continues during subsequent cycles by forming an island-like layer. Eventually, a dense layer is formed with a mosaic structure composed of larger inorganic patches embedded in a matrix of organic compounds. While the mosaic model for the structure of the SEI is generally accepted, our observations document in detail how the complex structure of the SEI is built up during discharge/charge cycling.

## Introduction

The solid electrolyte interphase (SEI) forming at the interface between the anode and the electrolyte is a key-enabling component of a lithium-ion battery and thus contributes essentially to the commercial success of the lithium-ion battery technology.^1−7^ The SEI allows the cells to be charged to high cell voltages exceeding the width of the electrochemical stability window of the electrolyte. The term SEI was proposed by Peled in 1979 implying that the ideal SEI should possess (1) negligible electronic conductivity to prevent further electrolyte decomposition and thereby passivate the interface and (2) high lithium-ion conductivity to guarantee lithium-ion transport across the layer.^8^ Originally proposed for alkali-metal anodes, the SEI concept was generalized to carbonaceous anodes and later to other anodes and more recently to cathodes, then often called a cathode (solid) electrolyte interphase (CEI).^9−11^

The formation of a passivating SEI during the first few discharge/charge cycles is a critical step in cell manufacturing and is carried out typically by the cell manufacturers to guarantee batteries with long cycle and calendar life and high safety.^12−15^ Consequently, the SEI is often named “the most important but least understood” component of a lithium-ion battery.^16^ The SEI basically consists of organic and inorganic electrolyte decomposition products. Cell manufacturers often employ proprietary electrolyte formulations with blends of multiple sacrificial electrolyte additives to modify the composition, structure, transport, and mechanical properties of the SEI, but without a clear mechanistic understanding how they influence the SEI formation.^17^

Structural models for the SEI have evolved from a simple passivating film of few nanometers in thickness^8^ to a variety of layered and mosaic models,^3^ where the organic components penetrated by the liquid electrolyte are believed to be responsible for the lithium-ion transport and the inorganic components for the passivating properties of the SEI. It is important to keep in mind that the SEI is neither static in composition nor in structure but evolves and ages dynamically as a function of the applied discharge/charge protocol. Apart from the SEI components that precipitate on the electrode surface, electrolyte decomposition products which dissolve in the electrolyte or evolve as gases need to be considered as well.^16,18^

The complexity arising from the spatial and temporal dynamics of the SEI during discharge/charge cycling demands for advanced operando measurements on the nanoscale.^19,20^ Many studies reporting on the composition and structure of SEIs rely on post mortem and ex situ SEI characterization but inevitably introduce significant damage to the SEI during electrode extraction, preparation, and handling. In particular, removal of the electrolyte readily introduces irreversible modifications to the SEI composition and structure and the SEI tends to by highly sensitive to exposure to ambient air.^21^

Recently, a number of operando techniques have been developed and applied to the study of the SEI growth with the goal to mitigate/minimize SEI damage, including Fourier transform infrared and Raman spectroscopy, X-ray-based methods, nuclear magnetic resonance, atomic force microscopy, and acoustic methods, but also electron microscopy methods.^22−38^ By employing a specially designed electrochemical liquid cell for in situ S/TEM, Zheng et al. were able to image the SEI formed on a lithiated gold electrode.^39^ Another in situ TEM study conducted by Unocic et al. revealed that an SEI formed on the gold electrode prior to Li plating, which remained on the surface after Li stripping.^40^ Yet, none of the previously mentioned studies, using different techniques, uncovered the exact nanoscale mechanism of the SEI growth in real time and operando, i.e., during discharge/charge cycling. Therefore, very little is known about the nanoscale evolution of the SEI during the first few cycles.

Here, by using an electrochemical liquid cell in scanning transmission electron microscopy, we study operando and at high spatiotemporal resolution the growth and evolution mechanisms of the mosaic solid electrolyte interphase on a glassy-carbon working electrode immersed in a classic electrolyte for lithium-ion batteries consisting of 1 M LiPF_6_ in ethylene carbonate/ethyl methyl carbonate (EC/EMC) under applied cyclic voltammetry (CV). We observe in real time how the SEI formation is initiated by the nucleation of inorganic nanoparticles, which grow in size until island-type regions start to form on the anode, which cross-link and connect during discharge/charge cycling. With further cycling, a continuous and dense SEI layer is formed on the glassy-carbon electrode, which prevents further electrolyte decomposition.

## Results and Discussion

### Assembly of in Situ Electrochemical Cell

**Figure 1:**
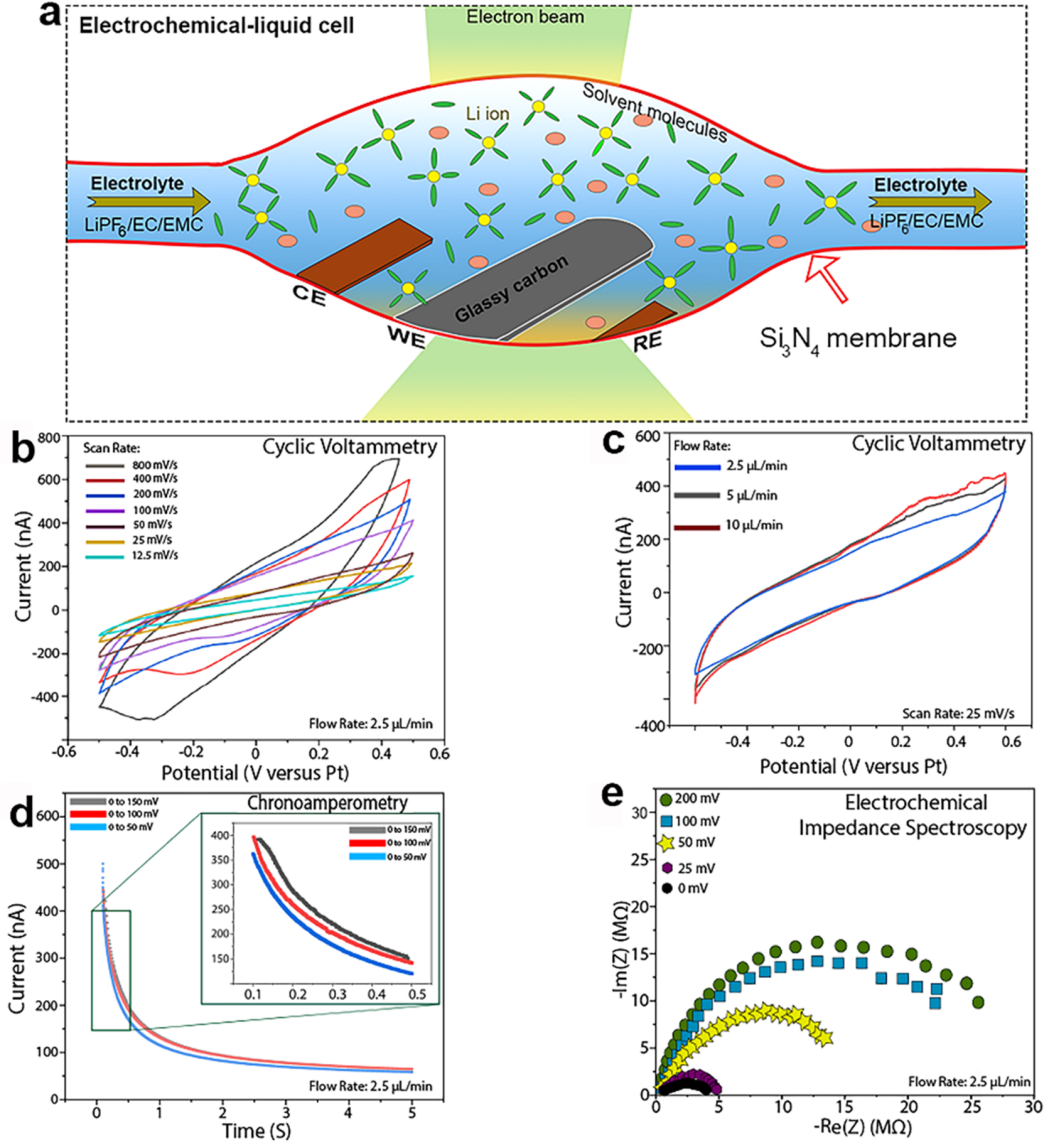
In situ electrochemical liquid cell for TEM/STEM and typical electroanalytical measurements performed for a [Fe(CN)_6_]^3-/4-^ redox couple: (a) cross-sectional illustration of in situ ec-LC S/TEM; (b) example of typical CV curves at different scan rates; (c) example of typical CV curves at different electrolyte flow rates; (d) example of typical CA for different potential ranges; (e) Nyquist plots at different potentials.

Figure 1a shows a schematic illustration of the in situ electrochemical liquid cell used to observe in real time the electrochemical reactions by STEM (more details about the liquid cell setup can be found in the Supporting Information). This design allows operando electrochemistry experiments to be performed while imaging at high spatiotemporal resolution the growth and evolution of the solid electrolyte interface.

The electrochemistry experiments performed in our study are in a half-cell configuration, where the liquid organic electrolyte, composed of solvent molecules and the electrolyte salt, flows in and out and covers the electrodes to enable ion transport between them (Figure 1a). To check the feasibility of performing such operando experiments using this miniaturized liquid cell setup, we performed electroanalytical measurements such as cyclic voltammetry, chronoamperametry (CA), and electrochemical impedance spectroscopy (EIS) within the cells for a [Fe(CN)_6_]^3–/4–^ redox couple. Figure 1b,c shows a typical measurement of cyclic voltammetry performed using our in situ ec-LC-S/TEM setup. The cyclic voltammetry versus scan rate (Figure 1b) shows that with increasing scan rate at a fixed electrolyte flow rate of 2.5 μL/min, the current increases slightly. However, increasing the flow rate does not show any effect on the CV curves. This finding allowed us to choose a flow rate of 2.5 μL/min and a scan rate of 100 mV/s in all of our experiments for consistency. EIS and CA studies are plotted in Figure 1d,e, respectively. EIS reveals the electronic resistance. Particularly, the EIS arc radius is related to the electronic resistance and transfer among the working electrode and the interfaces. CA is used to study the performance of the electrodes and the electrolyte. Thus, the CA and EIS measurements presented in Figure 1d,e demonstrate the feasibility of operating this electrochemical cell and the good contact between the electrodes and the liquid electrolyte.

Since the reference electrode in our electrochemical cell is made of platinum, we calibrated the potential from the Pt reference (pseudo potential) to Li/Li^+^ (see Methods and Figure S4 for more details) in order to make the subsequent experimental measurements consistent and comparable to previous studies.

### Formation of SEI Layer

**Figure 2:**
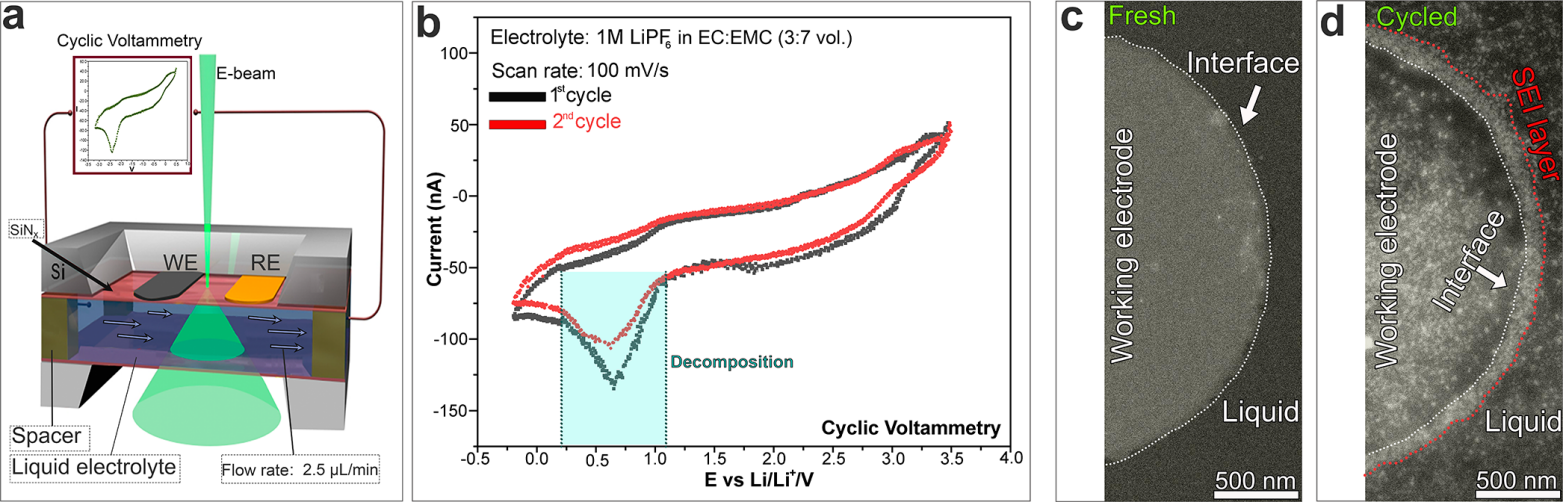
In situ electrochemical liquid cell STEM visualization of the GC–electrolyte interface during cycling: (a) schematic illustration of the ec-LC-STEM setup used for electrochemical measurements, illuminated with a scanning electron beam and connected to an external potentiostat for performing the electrochemical measurements; (b) cyclic voltammograms obtained from the operando ec-LC-STEM cell showing charge–discharge from the GC electrode in LiPF_6_/EC/EMC liquid electrolyte at a flow rate of 2.5 μL/min and cycled with a scan rate of 100 mV/s; (c, d) two typical ADF-STEM images of the GC–electrolyte interface before and after the first few cycles.

To study the formation of the SEI layer, we carried out successive voltammetry cycles of the cell studied by in situ ec-LC S/TEM, using a commercial electrolyte containing 1 M LiPF_6_ in EC/EMC (3:7 by volume). Since it is well accepted that LiPF_6_/EC/EMC electrolytes have a decomposition reaction potential of around 0.8 V versus Li/Li^+^, we chose to cycle our system in the voltage range of 3.5 to −0.25 V versus Li/Li^+^ (corresponding to 0.5 to −3.5 V versus Pt).^41^Figure 2a shows a detailed schematic illustration of the in situ ec-LC S/TEM setup used for our experiment, illuminated with a scanning electron beam, depicting the connection to the external potentiostat used to perform the CV measurements. Figure 2b shows two typical successive cyclic voltammetry curves of the cell, filled with 1 M LiPF_6_ in an EC/EMC electrolyte. During the first lithiation (black line), one redox peak appears at around 0.75 V versus Li/Li^+^. This peak corresponds to the irreversible decomposition of the electrolyte and the initiation of the SEI layer formation on the carbon surface.^41^ During the second CV curve, the peak at 0.75 V is considerably suppressed (decreased by about a factor of 2 (red line)), suggesting that the initial SEI layer has already been formed while the electrolyte further decomposes and the SEI continues growing. This means that the SEI layer formed during the first CV cycle is incomplete and that the electrolyte is still in contact with the glassy-carbon (GC) electrode, which leads to further decomposition.

Figure 2c shows a typical annular dark-field scanning transmission electron microscopy (ADF-STEM) image of the interface between the glassy carbon and the electrolyte before cycling, while Figure 2d shows a typical ADF-STEM image of the same region (interface) after the first few CV cycles. Interestingly, before cycling, the interface appears to be smooth (highlighted by a white dashed line). However, after a few cycles, a SEI layer with an inhomogeneous structure is formed, as highlighted by the red dashed line in Figure 2d.

### Operando Nucleation and Growth of SEI layer

**Figure 3:**
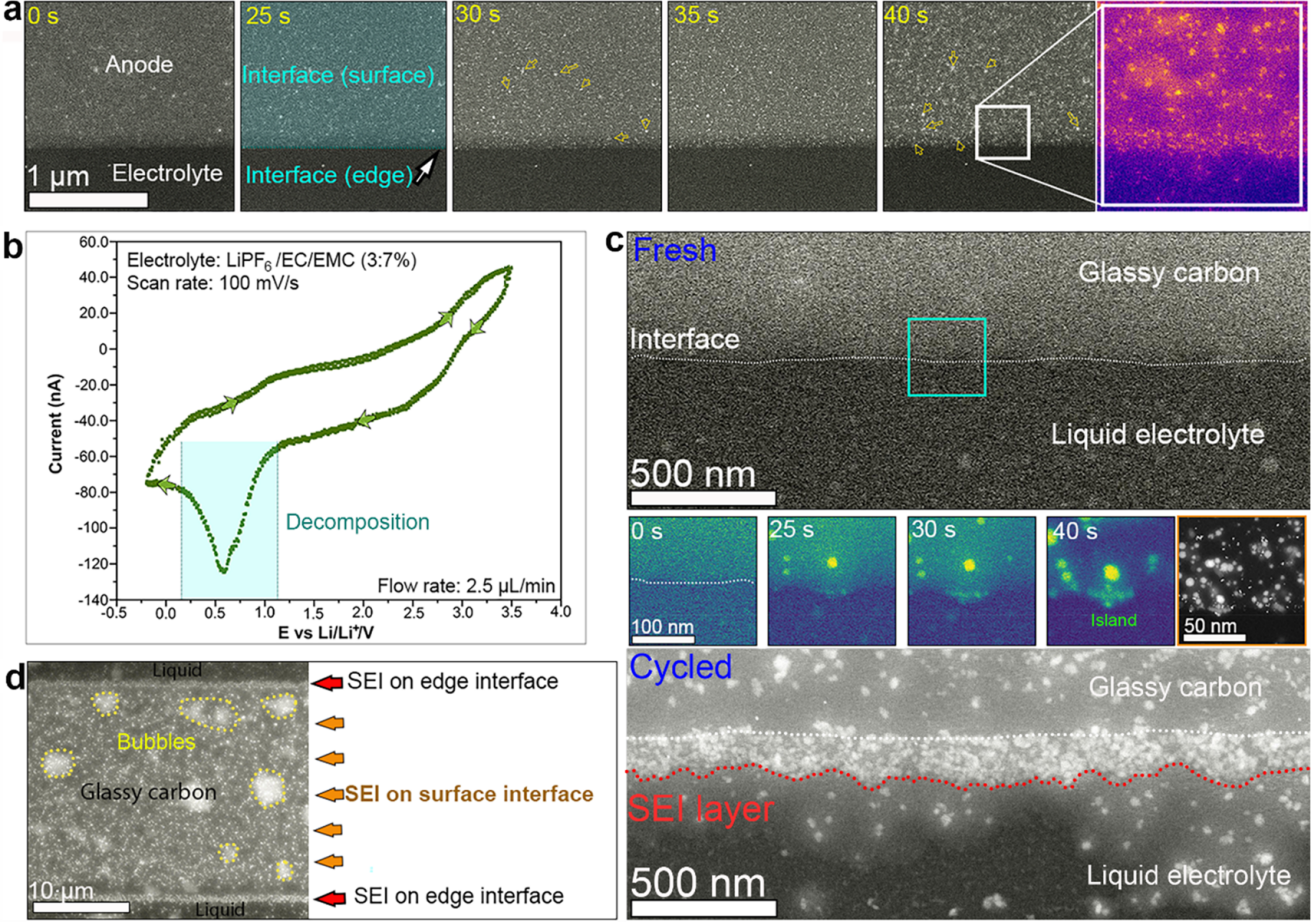
Time evolution of the GC–electrolyte interface during cycling: (a) time lapse series of ADF-STEM images of the GC-electrolyte interface showing the growth of SEI during cycling; (b) cyclic voltammogram obtained from an operando ec-LC-STEM showing charge–discharge from GC electrode in 1 M LiPF_6_ /EC/EMC liquid electrolyte at a flow rate of 2.5 μL/min; (c) ADF-STEM images (top, anode–electrolyte interface before cycling; bottom, anode–electrolyte nterface after cycling; middle, time-lapse series of ADF-STEM images of the edge of GC showing the growth of the SEI layer during cycling); (d) ADF-STEM image showing an overview of the GC electrode after a few cycles, where an SEI is clearly visible, and bubbles are formed.

To unveil the exact pathway of the SEI formation depicted in Figure 2d, we monitored on the nanoscale and in real time the GC–electrolyte interface and its surrounding area during cycling. In particular, we imaged the interface exactly in the potential range 0.2–1.15 V versus Li/Li^+^, within which the irreversible redox peak at ∼0.75 V is observed (highlighted by the turquoise rectangle in Figure 3b).

Figure 3a shows sequential ADF-STEM images from movie S1, representing the early stages of the electrolyte decomposition and the evolution of the GC–electrolyte interface during cycling (Figure 3b). Based on the CV curve in Figure 3b, the decomposition of the electrolyte occurs at a voltage range between 1.15 and 0.2 V versus Li/Li^+^. The time *t* = 0 s is the time of the beginning of the cycling, starting at 3.5 V versus Li/Li^+^ with a scan rate of 100 mV/s. Between *t* = 25 s and *t* = 30 s, we start observing small bright dots on the surface and at the edge of the GC electrode (indicated by yellow arrows in Figure 3a), which can be attributed to decomposition of the LiPF_6_ salt in the electrolyte. Thereafter, between *t* = 30 s and *t* = 40 s the growth continues, where the number and size of particles increase to form small regions, and then a large bright layer is formed at the surface and at the edge of the GC electrode. Indeed, the enlargement in Figure 3a (false color), corresponding to the region highlighted by the white square at *t* = 40 s, shows clearly the formation of a thin SEI layer at the edge of the glassy carbon as well as at the surface. The formed solid interface, both at the surface and at the edge of the GC, is based on small island-like patches.

In order to understand the nucleation and growth mechanisms of the SEI layer, we imaged at high spatial resolution a small zone from the edge and the surface of the GC in contact with the liquid electrolyte during electrochemical cycling. We focused our observations on the small area highlighted by the turquoise square in Figure 3c. At the beginning of cycling (*t* = 0 s) the interface between the GC and the liquid electrolyte (highlighted by a white dashed line in Figure 3c-fresh) appears to be smooth. After 25 s of cycling, a batch of small nuclei has formed, observable by bright contrast. Here, the ADF-STEM images are used in false color to highlight the contrast between the nanoparticles and the GC. After 30 s, the SEI nuclei have grown further via joining of adjacent nuclei and forming of an island-like porous structure, which partially covers the GC edge. When the discharge process continued, as the voltage was further decreased from 0.56 to 0.15 V (*t* = 30 to 34 s), the decomposition of the electrolyte occurred at the GC surface. When zooming out at the end of the first cycle, clear island-like regions are visible at the edge and at the surface of the GC electrode (also see movie S2 and Figure 3c-middle highlighted by brown squares), also indicating that electron-beam effects are minimal. After 3 cycles, a distinct continuous layer is formed at the edge of the GC. At this stage, the formed SEI layer has an approximate thickness of 160 nm (highlighted by a red dashed line in Figure 3c-cycled). The ADF-STEM image in Figure 3d provides an overview of the GC after 3 cycles. One can clearly see that an SEI layer has built up at the edge and on the surface of the GC. In addition to the SEI, tiny bubbles show up (highlighted by yellow dashed lines), indicating the generation of gaseous products during cycling, likely CO_2_^18^ and/or PF_5_.^42^ Our observations of the formation and growth process of the SEI layer is consistent with the phenomenological model proposed by Zhang et al. and the mechanisms described by Sundararajan et al.^43,44^ One can see also that bright dots are appearing in the liquid area surrounding the GC electrode. This can be attributed to the detachment of nanoparticles from the large surface of the GC electrode or also can be attributed to organic compounds that can dissolve partially in liquid (more details are given in Figure S5).

In order to be sure that the generated species for the SEI and the bubbles are formed by electrochemical cycling and are not caused by electron beam irradiation by 300 keV electrons, we also performed control experiments, and the results show that without applied cyclic voltammetry, neither the aforementioned bubbles form nor precipitation of lithium occurs (see Figure S3). Although electron-beam effects are complex and difficult to quantify, our extensive experiments and control experiments show that beam effects are negligible in our studies. The nanoparticle growth and SEI formation only occur under cyclic voltammetry in the electrochemical liquid cell.

### Evolution of SEI Layer

**Figure 4:**
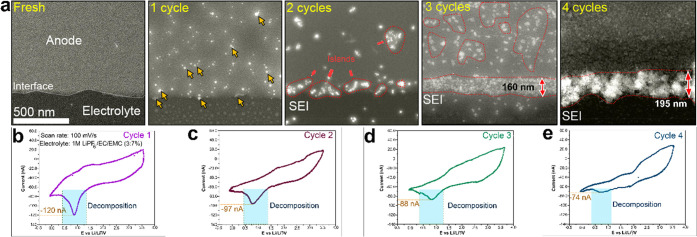
Evolution of the SEI layer with increasing number of cycles: (a) time series of ADF-STEM images showing the evolution of the SEI at the glassy-carbon electrode; (b–d) first, second, third, and fourth CVs, respectively, obtained from an operando ec liquid cell STEM experiment showing charge–discharge from GC electrode in LiPF_6_/EC/EMC liquid electrolyte at a flow rate of 2.5 μL/min.

After studying the nucleation and growth mechanism of the first product of the SEI layer during the first cycle, we aimed at uncovering the exact mechanism of its evolution with an increasing number of cycles. Figure 4 shows comparative CV curves of the first four cycles and corresponding ADF-STEM images of the interface between the GC and the electrolyte, performed in a potential range of 3.5 to −0.25 V vs Li/Li^+^. During the first CV cycle (Figure 4b), nanoparticles nucleate on the surface and at the edge of the GC during discharge (indicated by yellow arrows). The SEI nucleation observed earlier occurs at the irreversible peak at 0.75 V corresponding to electrolyte decomposition. Interestingly, during the second CV cycle (Figure 4c), the 0.75 V peak is still observable but with reduced intensity, namely, −97 nA compared to −120 nA in the first cycle. Regarding the morphology and the structure of the SEI layer, a clear evolution can be recognized between the first and the second cycle. When looking at the growth of nanoparticles that form island-like regions on the surface and the edge-interface, as indicated by dashed red lines, one can appreciate that the particle size and the concentration of the SEI layer are higher at the edge-interface. Likewise, at the third CV cycle, the intensity of the 0.75 V peak decreases further to −88 nA (Figure 4d), while the thickness of the SEI layer further increases. The island-like regions coalesce to form a continuous layer of about 160 nm thickness at the edge, and a larger island-like SEI layer at the surface is established, as indicated by dashed red lines. After four cycles, the decomposition peak is almost flat (Figure 4d), indicating that the electrode is passivated and the SEI forms a continuous compact layer with a thickness of ∼200 nm, which is consistent with previous studies.^39^ We speculate that the large SEI obtained in the ec-STEM cell originates from the presence of orders of magnitude more electrolyte per active surface area relative to typical commercial batteries and current field lines are being concentrated at the electrode’s edge. Also, the surface of the GC is completely covered by a continuous SEI layer, which prevents further decomposition and growth of the SEI. The formed SEI layer is highly porous, which can be attributed to the presence of a high concentration of organic compounds, which provide little image contrast (see Figure S6). Figure 4a also shows that the SEI layer, after four cycles, is composed of regions having different contrasts, which confirms the mosaic structure of the formed SEI layer. Therefore, the observations reveal that during the first CV cycle, the SEI layer nucleates while it undergoes growth and densification during the following cycles. Importantly, after formation of the SEI, the cell was kept under a flow of 2.5 μL/min, without significant changes in SEI structure or thickness, when imaging again after 40 min (Figure S7), contrary to the beginning of the growth of the SEI where we could observe detachment of some particles from the surface of the GC. In order to perform a chemical analysis and to record high-resolution ADF-STEM images of the SEI layer, after 4 cycles the electrochemical microchips were transferred to a glovebox where they were washed with dimethyl carbonate (DMC) for an ex situ analysis (see the Methods section for more details), and then transferred back to the TEM using a chip inspection holder. One can consider the formed layer as Li metal instead of an SEI layer; however, using the ADF-STEM imaging technique, the contrast is atomic number sensitive, where the intensity is proportional to the atomic number (*Z*) (∼*Z*^1.82^), and for lithium *Z* = 3 and for electrolyte *Z* ≈ 6–8, then the Li should appear as dark on a bright GC background.

### Nanoatomic Structure and Chemical Composition of the SEI Layer

**Figure 5:**
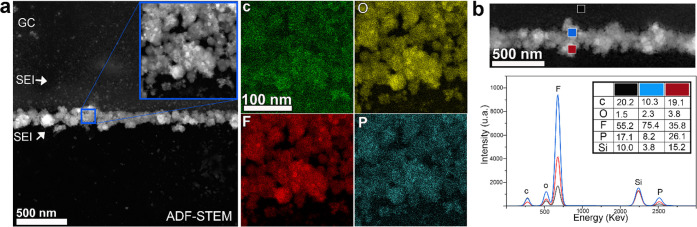
STEM/EDS analysis of the SEI layer: (a) ADF-STEM image of the formed SEI layer after 4 cycles with EDS maps of C, O, F, and P corresponding to the area in the blue square; (b) ADF-STEM image of the SEI with corresponding EDS point analyses indicated by different colors.

We have conducted energy dispersive X-ray spectroscopy (EDS) mapping to study the elemental distribution at a small, but representative, region of the formed SEI layer after four cycles (highlighted with a blue square in Figure 5a). The EDS measurements were carried out ex situ, after carefully removing the liquid electrolyte and gently cleaning the electrochemical microchips inside a glovebox in order to get rid of the organic residues (more details in the Supporting Information). The EDS maps displayed in Figure 5a provide elemental distribution information, where a homogeneous distribution of C, O, F, and P can be noticed. No electron-beam-induced damage is visible. The ADF-STEM image combined with EDS mapping indicates that the SEI layer is formed by an agglomeration of nanodomains separated by antiparticle voids and grain boundaries. Thus, a distinct mosaic structure is confirmed. The elemental composition was studied by using EDS point analysis at different locations of the SEI layer (Figure 5b). The surface region (black square) shows a significant concentration of carbon due to the vicinity of the glassy-carbon surface. However, the concentration of carbon decreases in the inner part of the SEI (blue square), while that of oxygen and fluorine increases. This can be related to the coexistence of inorganic (LiF, Li_2_CO_3_, and Li_2_O) and organic (ROLi, ROCOOLi, and RCOOLi) compounds.^41^ It thus appears that a higher concentration of the inorganic compounds is found in the inner part whereas the organic compounds dominate in the outer part of the SEI layer.^42^ Moreover, this chemical analysis also supports the model of the mosaic nature of the formed SEI layer. We also used scanning electron microscopy (SEM) to compare the surfaces of the GC electrode before and after four cycles. Indeed, the SEM images in Figure S8a,b show that the smooth surface and edge of the GC before cycling is covered after four cycles and that a distinct SEI layer is formed at the edge of the GC. The detail depicted in Figure S8d confirms the mosaic nature of the SEI layer. Interestingly, using EDS analysis in SEM we were able to detect lithium all over the SEI layer as well as other elements such as F, O, C, and P, which can indicate the presence of LiF and Li_2_O compounds (Figure S8c).

The increase in the concentrations of C and O at the outer part of the SEI can be attributed to the increasing amount of organic compounds. The homogeneous distribution of P in the three regions of the SEI can be attributed to the presence of residual LiPF_6_ salt on the surface of the glassy carbon and on the SEI layer.

**Figure 6:**
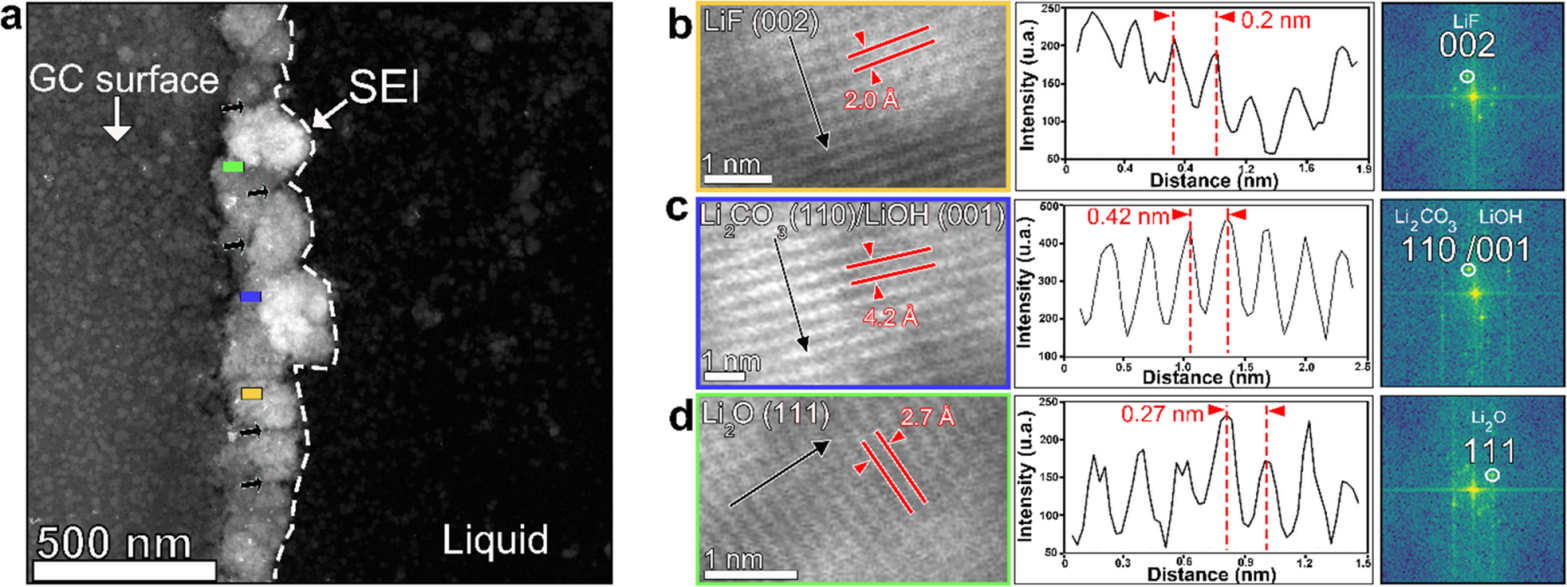
Identification of SEI compounds by high-resolution ADF-STEM imaging, Fourier transforms, and lattice spacing: (a) ADF-STEM image of the SEI after the first few cycles; (b) lattice spacing for LiF (002) and corresponding intensity profile and FFT; (c) lattice spacing of Li_2_CO_3_ (110) or LiOH (001) and the corresponding intensity profile and FFT; (d) lattice spacing for Li_2_O (111) and the corresponding intensity profile and FFT.

In order to study the atomic structure of the compounds forming the SEI layer, we performed high-resolution ADF-STEM (HR-ADF-STEM) imaging. Because lithium in particular and the SEI in general are very sensitive to electron beam irradiation, we used a very low electron dose rate and acquired HR-ADF-STEM from the same microchip employed for the EDS analysis. From the corresponding fast Fourier transforms (FFTs) we determined the atomic structure of the crystalline phases. At different regions of the SEI layer formed after four cycles, HR-ADF-STEM images were collected, and three types of lattice fringes were detected. Lithium fluoride (LiF), lithium oxide (Li_2_O), and lithium carbonate (Li_2_CO_3_) and/or lithium hydroxide (LiOH) were identified from three different regions of the SEI (marked as pink, blue, and red squares, respectively, in Figure 5a), which is in agreement with the elements detected using EDS analysis. In the region marked by the pink square, the *d* spacing was measured to be 2.0 Å (Figure 5b), which corresponds to the (002) lattice spacing of LiF. For the region marked by the blue square, the *d* spacing was measured to be 4.2 Å, which corresponds to the (110) lattice spacing of Li_2_CO_3_ or to the (001) lattice spacing of LiOH, and for the region marked by the red square, the *d* spacing was measured to be 2.7 Å, which corresponds to the lattice spacing of Li_2_O (111). This confirms the coexistence of the inorganic compounds previously reported that are supposed to dominate the inner part of a mosaic SEI layer.^41^ The dominant presence of inorganic compounds in the inner part confirms that the initially formed species are inorganic compounds. Figure 6a shows that the formed SEI layer still possesses a porous nature, which can be attributed to the presence of organic compounds (highlighted by the black arrows in Figure 6a). In order to compare the formed NPs at the beginning of the growth to those forming the final SEI, we imaged the formed nanoparticles after two cycles by HR-ADF-STEM imaging (see Figure S9), and indeed, we found some nanoparticles corresponding to LiF and Li_2_CO_3_, which indicate that the inorganic nanoparticles formed at the beginning of the cycling are the same forming the mature dense SEI layer.

Despite notable achievements in revealing the SEI structure and composition, since its proposition by Peled in 1979,^8^ still little is known about its nucleation, growth, and formation process. However, recently progress has been made using in situ and ex situ techniques to shed light on the growth and structure of SEI layers. For example, Zheng and co-workers studied the SEI growth in a commercial electrolyte by operando liquid cell TEM. They demonstrated that the reduction of electrolyte induces the growth of an SEI layer, where an initial fast growth occurs during the first cycle. When the voltage is decreased to 0 V, the growth rate drops to almost zero.^45^ Moreover, it was shown in the study that the SEI layer is formed on the surface of the active electrode until a thickness of about 200 nm is reached and maintained. This is due to the low electron mobility, which causes limited growth of the SEI.^39^ Moreover, Sundararajan et al. used a combination of in situ and ex situ characterization tools to study the formation process of the SEI layer, where they evidenced its mosaic structure. Notably, ex situ TEM characterization revealed the evolution of the SEI layer during the first lithiation. Furthermore, the study showed that the SEI formation is initiated by nucleation and growth of nuclei, followed by the formation of island-like structures on the graphite surface. After a first discharge, a continuous SEI layer is formed with a mosaic structure. Thus, a mosaic structured SEI layer is confirmed and shown to be composed of inorganic and organic compounds.^33^ Yet, none of these studies discussed the exact mechanisms of the nucleation and growth processes, and none of these studies shed light on how a continuous dense mosaic SEI layer is formed with the primary species of nucleation, and how the SEI progresses with increasing number of cycles. The present study goes beyond the previously suggested mechanisms, since we track in real time the nucleation and growth of the SEI compounds to uncover the exact formation mechanisms of the mosaic SEI layer. Interestingly, we reveal the exact evolution pathway of the SEI layer with cycling.

**Figure 7:**
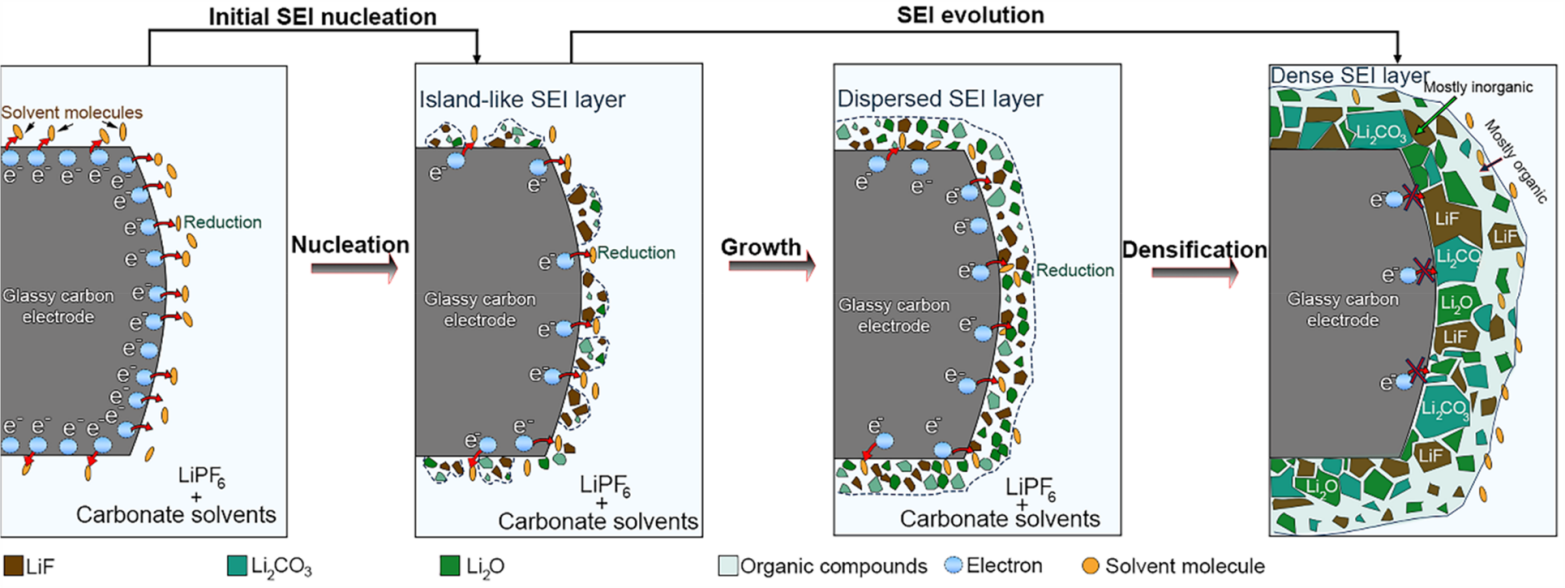
Schematic representation of the formation pathway of the SEI layer generated on a glassy-carbon electrode. The formation is divided into two main steps: (I) initial SEI nucleation and (II) SEI evolution.

According to our operando observations, the mechanism governing the formation of the mosaic SEI layer appears to be more complex than expected and comprises multiple steps. Our real-time observations reveal that this mosaic structure is formed via two main steps: the first step is called “initial “SEI nucleation”, where the electrolyte is reduced to form an island-like layer. The second step is called “SEI evolution”, in which the SEI layer matures by growth and densification processes, as illustrated in Figure 7.

Our study shows that the reduction of the electrolyte containing LiPF_6_, EC, and EMC occurs at negative potential, at which an irreversible peak at 0.75 V versus Li/Li^+^ is observed, which allows the formation of ultrasmall inorganic species, i.e. nanoparticles observed with bright contrast in ADF-STEM. This is in agreement with previous studies.^43^ Due to the high contrast and the stability of the formed nanoparticles under the electron beam, in addition to the structural investigation, it has been demonstrated that these primary species are mostly inorganic compounds. This can be attributed to LiF compounds resulting from the decomposition of LiPF_6_ salt, which is demonstrated to be the first species that can be generated during cycling, based on previous studies.^2^ We considered this stage as the nucleation phase in SEI formation, as illustrated in Figure 7. In a second step, the species formed during nucleation increase in number and in size. This can be due to further decomposition of LiPF_6_ and precipitation of carbonates from the electrolyte solvent with lithium ions to form other inorganic compounds such as Li_2_CO_3_, Li_2_O, lithium alkali carbonates, and other organic compounds, such as ROLi and ROCO_2_Li. This process causes the transition from an island-like layer to a dispersed SEI layer (Figure 7).^2^ In this stage, the formed layer is highly porous and principally formed with species containing interparticulate voids and wide grain boundaries. Consequently, with this porous nature of the SEI layer, the electrolyte can still reach the surface of the GC electrode. This facilitates further decomposition to produce more species as cycling further continues. Especially, when the voltage decreases during the charge process in the following cycles, decomposition of the electrolyte occurs that generates new species, which will then cover more surface of the electrode (inner part), allowing the transition from a dispersed to a dense SEI layer. Thus, the formed SEI layer becomes continuous and dense, preventing further decomposition. This finding is supported by previous claims about the growth mechanism of SEI.^41^

The SEI formation process thus comprises two main steps. In the first step, during which the initial SEI formation takes place, the anode is polarized, and the species in the electrolyte undergo reductive decomposition, which allows the generation of inorganic compounds. In the second step, during which the SEI evolves further, the growth and coalescence of these nanoparticles represent the dominant process leading to a continuous SEI layer whereas the new compounds precipitate to form the SEI until the entire surface of the GC is covered with mostly inorganic compounds. When electrolyte decomposition is no longer possible through the dense SEI layer, an outer layer is established, which is dominated by organic compounds, while the inner layer will be dominated by inorganic species. It is still a mosaic structure, as the empty space between the inorganic compounds at the outer regions is filled by organic compounds. Thus, the final SEI layer is demonstrated to be porous indeed, with a compact layer of inorganic components (e.g., LiF, Li_2_CO_3_, and Li_2_O) close to the GC electrode followed by a porous organic layer (e.g., ROLi and ROCO_2_Li), which confirms the previous model of a mosaic SEI structure.^46^

## Conclusions

In this work, we have reported that fast imaging in STEM combined with in situ electrochemical liquid cell measurements provides a powerful tool to shed light on chemical reactions occurring inside batteries on the nanoscale. We monitored and controlled operando the very earlier stage of the nucleation of the SEI between a GC electrode and a classic 1 M LiPF_6_ in EC:EMC (3:7 volume) electrolyte. We identified that the growth of the mosaic SEI layer is governed by nanoparticle growth. We demonstrated that the growth of the mosaic SEI layer is a multistep process. By imaging the GC–electrolyte interface in situ, we show that the decomposition of the electrolyte at low potential results in the nucleation of inorganic nanoparticles. In this first step, nucleation of nanoparticles occurs during the first charge process, which is called initial formation. Then, in the second step, called SEI evolution, the island-like SEI layer transforms into a dispersed layer via a growth process, and then along with the deposition of organic components to a dense continuous layer via a densification process. Finally, a mosaic structured SEI layer is formed, which is composed of organic and inorganic compounds.

Our work demonstrates that this operando ec-S/TEM platform can be used to image locally in real time the growth process of the SEI layer during battery operation on top of a carbon-based electrode. This experimental approach can be adopted and extended to other systems, such as Li metal and Si anodes. In addition, the ec-S/TEM platform used here can be adapted and used to study the degradation and evolution of electrolytes as well as the evolution of cathode materials during the charge/discharge of the batteries. For each of the aforementioned examples that can be studied, the system needs to be adapted to fit with samples and the changes that can occur on the samples during battery operation. Future work will involve the investigation of the effect of electrolyte additives such as vinylene carbonate (VC) and fluoroethylene carbonate (FEC) on the formation mechanism and the structure of the SEI layer.

## Methods

### Materials

A solution of lithium hexafluorophosphate in ethylene carbonate and dimethyl carbonate (1 M LiPF_6_ in EC/EMC = 3/7 (v/v), 99.9% under argon) and bis(cyclopentadienyl)iron (Fe(C_5_H_5_)_2_) were purchased from Solvinic. Acetone, methanol, and dimethyl carbonate (anhydrous, 99%) were purchased from Sigma-Aldrich. All chemicals were used without further purification. Microchips (e-chips) for the electrochemical liquid cell were purchased from Protochips.

### In Situ Electrochemical Liquid Cell Setup

(1) Microfabrication techniques enable the production of microchips that contain electron-transparent Si_3_N_4_ viewing membranes, which are mechanically and chemically robust. Pairing such microchips allows enclosed cells to be built, wherein liquids can be studied in the vacuum environment of the microscope (Figure S1). Additional features such as working, counter, and reference electrodes with lithographically patterned spacers (e.g., SU-8 photoresist) provide electrical circuitry and isolation for electroanalytical measurements with connections to an external potentiostat.^47^ In addition, modern in situ TEM sample holders include integrated microfluidic tubing connected to an external syringe pump for controlled delivery and flow of electrolytes through the above-mentioned cell (see Figure S1 for more details).
(2) The in situ electrochemical liquid cell was assembled in the way shown in Figure S1, where two microchips are aligned on top of each other to form a closed cell with the help of an O-ring gasket sealing the liquid phase from the vacuum environment. The removable silicon microchip devices used in this study have a 50 nm thick amorphous Si_3_N_4_, electron-transparent window of 50 × 200 μm^2^. In addition to the silicon support frame and the silicon nitride membrane the small microchip (lower) has a 150 nm thick SU-8 photoresist material patterned on the corner of the e-chip (Figure S1), which acts as a spacer between the upper and lower microchips and controls the liquid layer thickness. The upper e-chip contains patterned electrodes, namely the Pt reference electrode (RE) and the counter electrode (CE). In addition, the Si_3_N_4_ viewing window supports glassy carbon (GC) acting as the working electrode (WE) and enables imaging the electrode–electrolyte interface (see Figure S1 for more details). Glassy carbon was specifically chosen as a model system for the WE because it can be deposited on the silicon nitride using standard microfabrication methods, it is inert to lithium intercalation, it will not degrade upon repeated cycling, and its use replicates typical carbon black additives used in commercial batteries. Moreover, the combination of being able to deposit a thin layer with controlled thickness 100–200 nm) and it being a weak electron scatterer (electron transparent) makes GC an ideal electrode for in situ SEI imaging.^48^ More importantly, GC is composed primarily of electrically conductive sp^2^-hybridized carbon, similar to graphite, and therefore intercalation of alkali ions does not occur; thus, any detectable contrast change during imaging through the cell will be due to the formation of a SEI and not due to the possible density expansion of the electrode because of Li-ion insertion.^49^

Figure S2 shows a schematic illustration of the in situ ec-LC S/TEM setup. Connections from the electrochemical cell to the potentiostat are made by wires that pass through the shaft of the TEM holder, whereas electrolyte is delivered with a microfluidic delivery system consisting of a syringe pump and poly(ether ether ketone) (PEEK) tubing (more details are given in Figure S2). To image the dynamics of electrochemical processes, electrochemical measurements are performed, while the working electrode–electrolyte interfaces can be imaged through the electrolyte and through the electron-transparent region of the electrochemical cell. The aforementioned design allows in situ electrochemistry experiments to be performed and the SEI growth to be imaged.

### In Situ ec-Liquid Cell Preparation and Optimization

(1) A solution of ferrocene (Fe(C_5_H_5_)_2_) was used to perform cyclic voltammetry, chronoamperametry, and impedance spectroscopy.
(2) For the cell preparation, two e-chips were used: the top e-chip with GC as the working electrode and Pt as the reference and counter electrodes (Figure S1). The bottom e-chip contains a spacer of 150 nm. After cleaning both of them using a conventional method (acetone 4 min and isopropanol 4 min, drying gently with air), both e-chips were deposited carefully (to avoid damage of the silicon nitride membranes) on top of each other to form a sealed cell of a thickness around 1 μm.
(3) The cell was mounted on a Protochips Poseidon holder as shown in Figure S1a. First, the holder was inserted in a vacuum station to test the cell under a high-vacuum atmosphere and then moved to the TEM. If the cell was properly prepared and there was no leak in the membrane, the next step was to connect the holder with the potentiostat and the pumping station, where a syringe, mounted on a syringe pump and containing the electrolyte, was connected to tubing of the holder.
(4) We injected the solution inside the cell at a very slow rate (2.5 μL/min) for 30 min. First, we calibrated the system and did open circuit measurements for 300 s. In order to optimize the imaging conditions we used an electron dose (∼5 × 10^2^ electrons/(Å^2^ s)) allowing enough resolution to image the interface while minimizing affecting the electrochemical measurements: electron beam effects are unavoidable in the electrochemical cell TEM experiments since an electron beam is necessary for imaging. However, beam effects can be minimized by reducing the electron dose. Figure S3a shows two typical cyclic voltammetry curves on the in situ cell filled with ferrocene (Fe(C_5_H_5_))_2_ with beam off and beam on. No apparent effect of the beam on electrochemical measurements was recognizable. Solvated electrons and free radicals can be generated as the electron beam passes through the electrolyte in the liquid cell. The solvated electrons can reduce Li ions in the electrolyte, which could introduce precipitation of Li in the electrolyte solution. However, under the imaging conditions we used with moderate electron current density (∼5 × 10^2^ electrons/(Å s)) no such effect was observed. Figure S3b shows typical ADF-STEM images of glassy carbon surrounded by the electrolyte, where under the aforementioned electron dose no Li precipitation is detected (after 10 min), that could potentially participate in the solid electrolyte interface formation.
(5) All cyclic voltammetry measurements were performed with Pt as the reference electrode (pseudo reference). In order to calculate the exact potential versus Li/Li^+^, we performed an experiment with a mixture of LiPF_6_ in EMC/EC (3:7 volume) and ferrocene (Fe(C_5_H_5_)_2_) (10 mmol/L). We performed cyclic voltammetry measurements (see Figure S4 for more details) with a scan rate of 100 mV/s to check at which potential (vs the reference electrode) the peaks of the Fc/Fc^+^ redox couple appear. The half-wave potential of the redox couple was then calculated as the average value of both peak potentials. We know from other experiments that this potential should be more or less 3.24 V vs Li/Li^+^. From this information, we estimated the vertex potentials of the CV scan for the SEI formation.
(6) The in situ and ex situ electron microscopy experiments were carried out using a FEI Titan Themis 80-300 S/TEM instrument with a probe Cs-corrector operated at 300 kV. ADF-STEM was routinely used, which provides a contrast approximately proportional to *Z*^*n*^ (with *n* = 1.6–1.8 and *Z* being the atomic number). Scanning electron microscopy analyses were performed using a Zeiss GeminiSEM 460 instrument with a Ultim Extreme EDS detector which allows for detecting Li. In STEM mode the electron dose was optimized such that the electron beam imaged the SEI growth with enough contrast but did not affect the electrochemical measurements (maximum ∼5 × 10^2^ electrons/(Å s)). The in situ TEM holder used for our experiment is a Poseidon 510 apparatus from Protochips. The potentiostat used for electrochemical measurements was a Gamry instruments reference 620 Potentiostat/Galvanostat/ZR.

### Sample Preparation and Optimization for ex Situ Characterization

After cycling and forming the SEI in situ, we removed the liquid electrolyte, while the cell was closed. In a second step the in situ holder was transferred to a glovebox, where the cell was disassembled, and we carefully collected the electrochemical e-chips in order to protect the membrane and the GC electrode. The e-chips were washed with DMC to remove the residual organic species and then placed on a Poseidon ex situ inspection holder from Protochips. The holder was then transferred in a plastic bag with an argon atmosphere to the microscope to be inserted and used for high-resolution and EDX analysis.

## Data Availability

The data that support the findings of this study are available from the corresponding author upon reasonable request.

## Supporting Information Available

Details for the sample preparation, in situ liquid cell and methods for analyses, and additional figures as described in the text (PDF)
In situ ADF-STEM observation, showing an overview of the SEI growth during the first cycle via nanoparticles nucleation (AVI)
In situ ADF-STEM observation, showing growth of SEI via formation of island-like layer (AVI)

## Supplementary Material

nn3c06879_si_001.pdf

nn3c06879_si_002.avi

nn3c06879_si_003.avi
