# Supplementary material for: Operando Electrochemical Liquid Cell Scanning Transmission Electron Microscopy Investigation of the Growth and Evolution of the Mosaic Solid Electrolyte Interphase for Lithium-Ion Batteries
Source: ACS Nano. 2023 Oct 13;17(20):20434–44. doi: 10.1021/acsnano.3c06879 (PMC10604081; doi:10.1021/acsnano.3c06879)
Supplement: Supplementary file 1 — nn3c06879_si_001.pdf [file nn3c06879_si_001.pdf]

Operando Electrochemical Liquid Cell Scanning Transmission Electron Microscopy Investigation of the Growth and Evolution of the Mosaic Solid Electrolyte Interphase for Lithium-Ion Batteries

Walid Dachraoui,*[1,2] Robin Pauer,[1] Corsin Battaglia,[2,3,4] Rolf Erni*[1,5]

[1]Electron Microscopy Center, Empa—Swiss Federal Laboratories for Materials Science and Technology, Überlandstrasse 129, 8600 Dübendorf, Switzerland.

[2]Materials for Energy Conversion, Empa—Swiss Federal Laboratories for Materials Science and Technology, Überlandstrasse 129, 8600 Dübendorf, Switzerland.

[3]Departement of Information Technology and Electrical Engineering—*ETH Zürich*, Gloriastrasse 35, 8092 Zürich, Switzerland.

[4]Institute of Materials – *EPFL*, Station 12, 1015 Lausanne, Switzerland.

[5]Departement of Materials—*ETH Zürich*, Wolfgang-Pauli-Strasse 10, 8049 Zürich, Switzerland.

*Corresponding authors:
Walid Dachraoui (walid.dachraoui@empa.ch)
Rolf Erni(rolf.erni@empa.ch)

Table of contents

Supplementary figures: Figures S1 to S9

Captions for movies: Movie S1 to S2

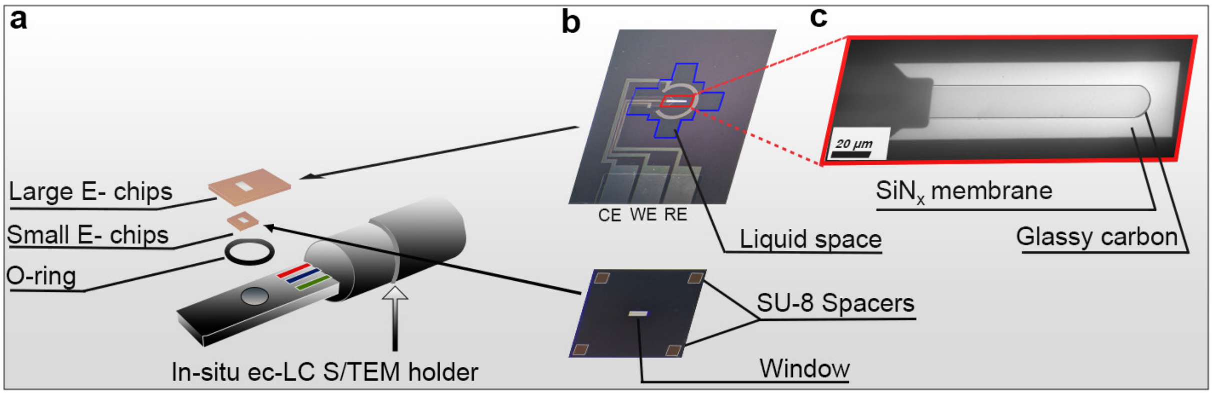

Figure S1. Schematic illustration of in situ ec-LC S/TEM cell. (a) Tip of Poseidon 510 TEM holder with precision slot for loading liquid cell, where the top microchip containing printed electrodes (working (glassy carbon), reference (platinum) and counter (platinum)) is mounted on top of a bottom microchip containing spacers. Both of them containing an electron beam transparent Si_3N_4 membrane, O-ring gasket is used to get a good vacuum-sealing. (b) Pictures of the electrochemical and space microchips. The microfabricated electrochemical chips contain coplanar working (WE), reference (RE), and counter (CE) electrodes that interface with an external potentiostat via electrical biasing wires and Pt microelectrode biasing contacts. The spacer maicrochip contains a spacer layer used to control the electrolyte layer thickness. (c) Scanning electron microscope images of the working electrode (Glassy carbon).

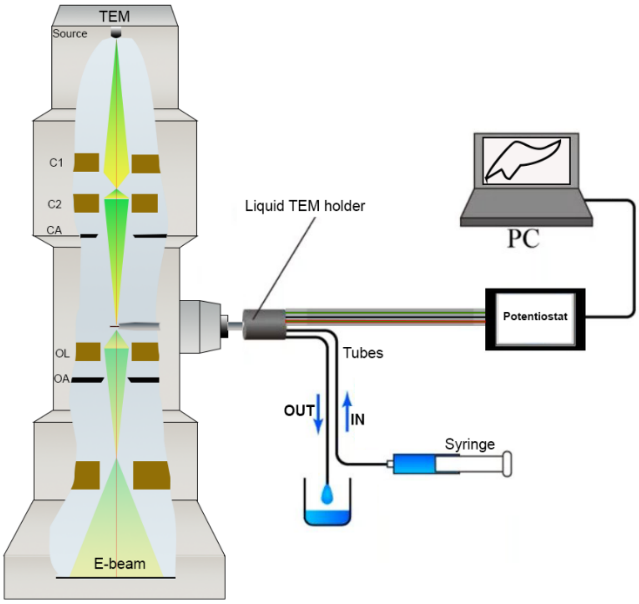

Figure S2. Schematic illustration of in situ ec-LC TEM setup. In situ liquid TEM holder connected to a potentiostat via special external cables, and to an external syringe pump connected to tubes, which insure the injection of the electrolyte inside the cell located in the TEM column.

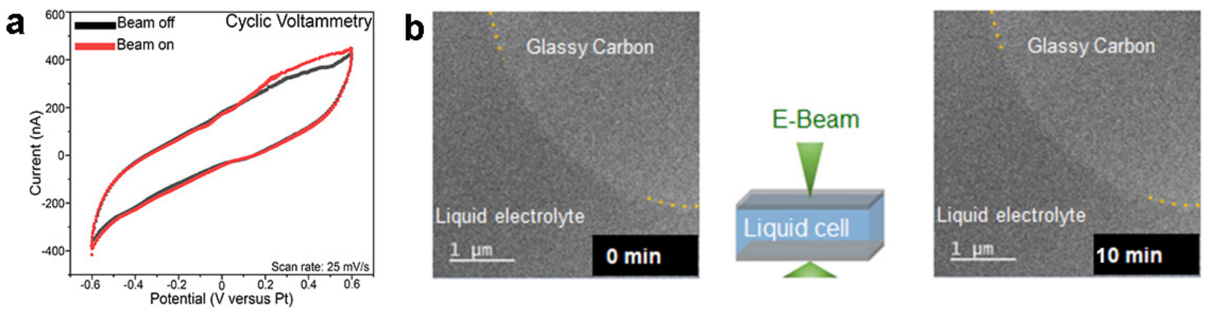

Figure S3. (a) Cyclic Voltammetry (CV) experiments with ferrocene Fe $(C_5H_5)_2$ having the beam off and beam on. (b) Glassy carbon electrode surrounded by liquid electrolyte exposed 10 min to e-beam. electron dose around 5 x 10^2 electrons/$Å^2$.s

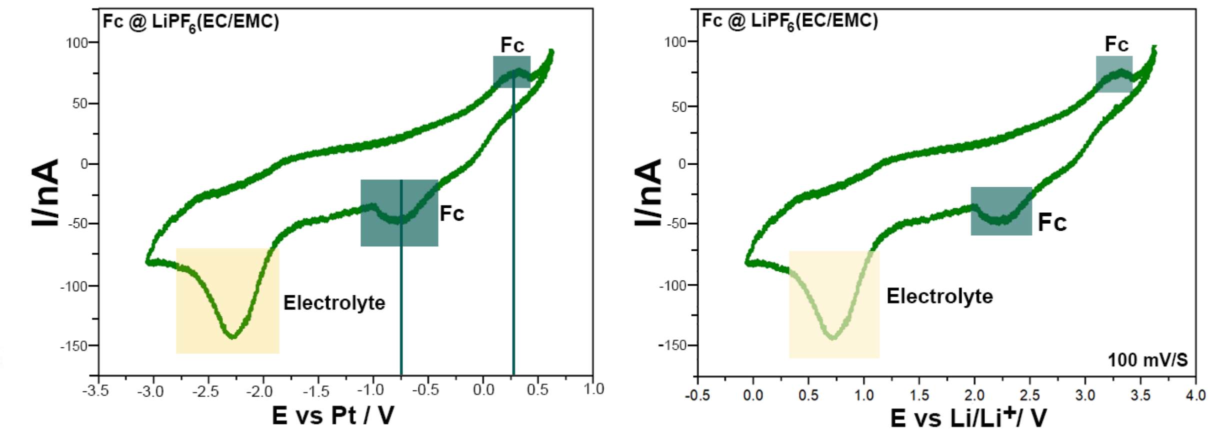

Figure S4. Cyclic voltammetry measurements of a mixture of $LiPF_6$ in EMC/EC (3:7 vol.) and Ferrocene Fe $(C_5H_5)_2$ used to calibrate the potential versus Li/Li^+.

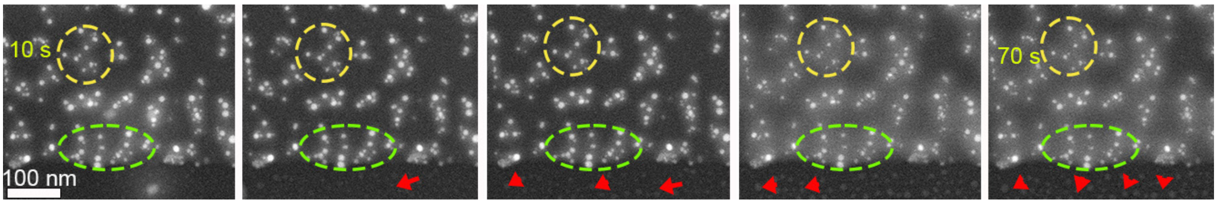

Figure S5. Time-lapse ADF-STEM image showing dissolution and detachment of NPs, highlighted by yellow and green dashed circles. Red arrows design the detached NPs.

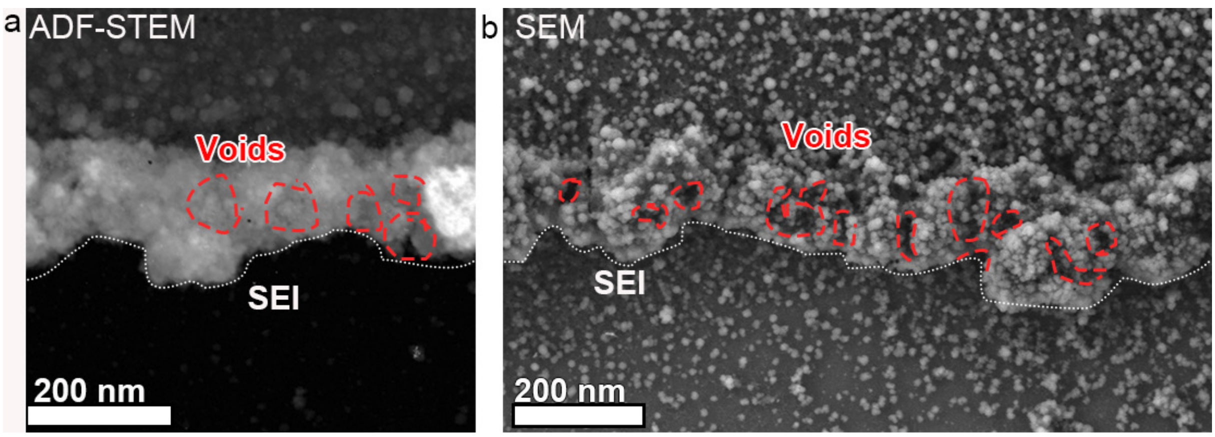

Figure S6. ADF-STEM and SEM image showing the SEI layer formed after four cycles with porous structure. (a) ADF-STEM. (b) SEM.

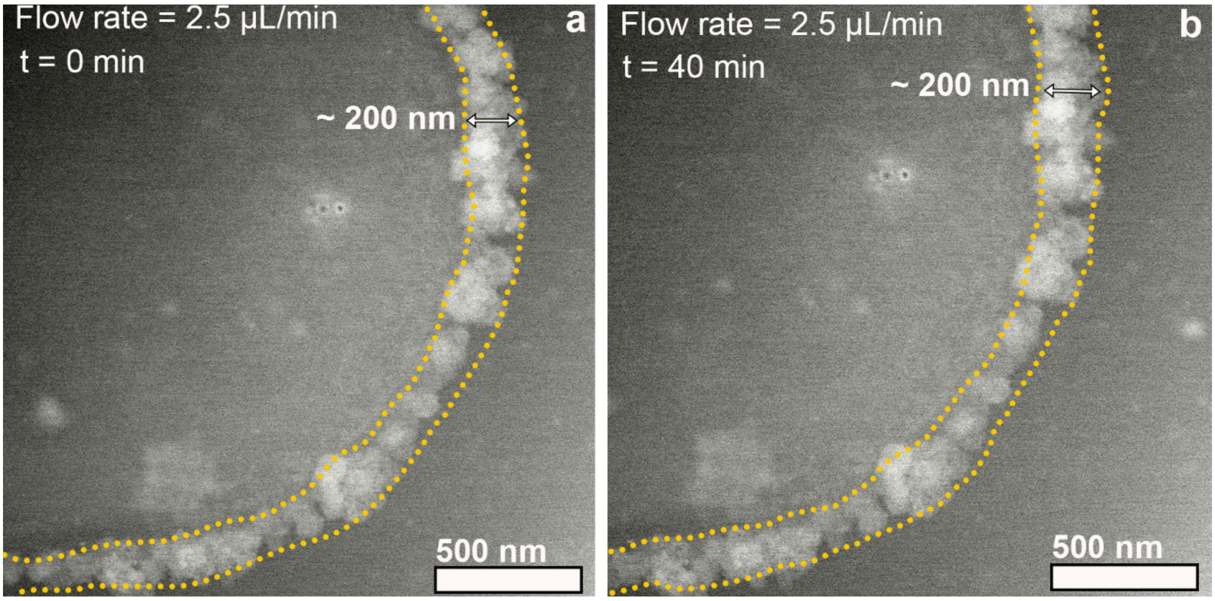

Figure S7. SEI layer formed after four CV cycles exposed to a continuous electrolyte flow. (a) SEI after completion of the formation cycles. (b) SEI after 40 min of electrolyte flow (flow rate of 2.5 μL/min, electrolyte volume 100 μL). t = 0 is the time just after the end of the fourth CV cycle.

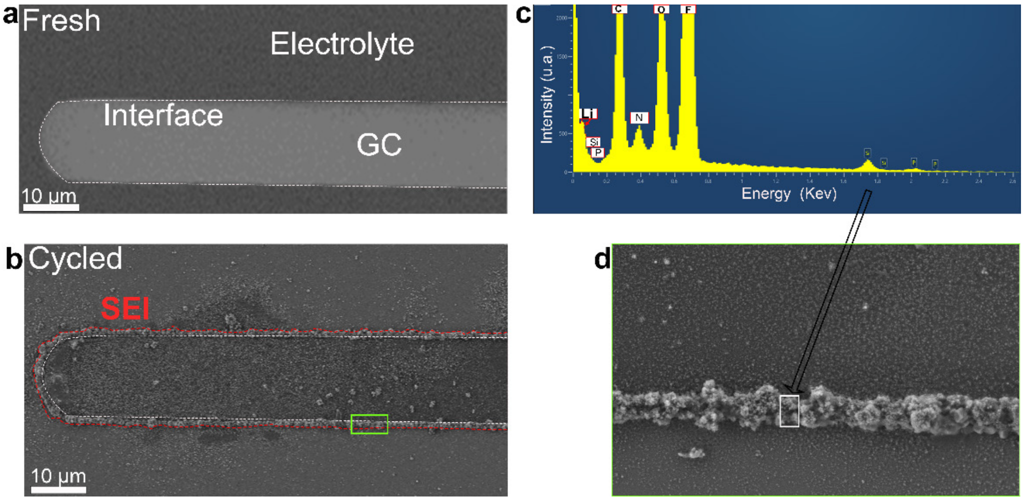

Figure S8. SEM image images showing the glassy carbon and the formed SEI after many cycles and corresponding EDX analysis. (a) Fresh GC. (b) Cycled GC with SEI layer. (c) EDX spectrum with lithium detection from region in (d). (d) Zoom in from the region highlighted with green rectangle in (b).

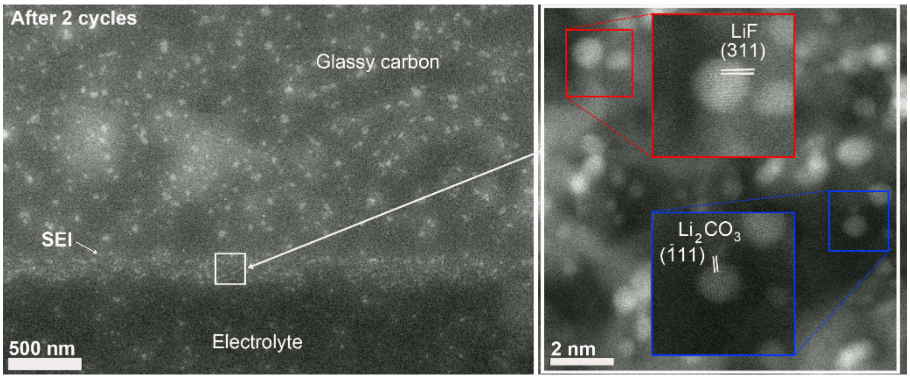

Figure S9. Identification of SEI first compounds by high-resolution ADF-STEM imaging. ADF-STEM overview image of the SEI after the first two cycles. (red) Nanoparticle with lattice spacing for LiF (311). (blue) Nanoparticle corresponding to Lattice spacing of Li_2CO_3 (-111).

Captions for movies

Movie S1: Overview of early stages of the electrolyte decomposition and the evolution of the GC-electrolyte interface during cycling with an applied voltage range of 0.2-1.15 V versus Li/Li^+. It shows the growth of nano-particles at the surface and the edge of GC electrode. Scale bar 500 nm.

Movie S2: Nucleation of the SEI layer at a small zone from the GC-electrolyte interface during cycling with an applied voltage range of 0.2-1.15 V versus Li/Li^+. It shows the growth of nanoparticles at the surface and the edge of GC electrode and the formation of island –like layer. Scale bar 30 nm.
